# Recurrent Unexplained Fever and Suspected Promazine Toxicity: Why Probable Neuroleptic Malignant Syndrome Was Left Undiagnosed in a Healthcare Worker

**DOI:** 10.7759/cureus.119209

**Published:** 2026-11-05

**Authors:** Simonas Makstutis, Suzy Collins

**Affiliations:** 1 Acute Internal Medicine, Stockport NHS Foundation Trust, Manchester, GBR

**Keywords:** diagnostic delay, healthcare professionals (hcp), medication toxicity, neuroleptic malignant syndrome (nms), promazine, recurrent fever

## Abstract

Neuroleptic malignant syndrome (NMS) is a potentially fatal adverse reaction to dopamine-antagonist medication that can resemble sepsis. A 51-year-old woman working in healthcare experienced recurrent episodes of fever, confusion and muscle rigidity, with six hospital presentations over approximately 10 months. Her prescribed medicines included promazine and amitriptyline. During an earlier severe admission, she developed hyperthermia, agitation, shock, and a creatine kinase concentration of 7272 U/L, requiring intensive care. Investigations did not identify a unifying diagnosis. During the most recent admission, review of previous records and a non-judgemental discussion elicited possible use of additional medication for sleep and neuropathic pain, although the preparation and quantity remained unclear. Six months' supply of promazine had been approved and dispensed within the preceding three months, supporting increased access without establishing the amount ingested. The clinical pattern supported a working diagnosis of probable promazine-associated NMS, with possible concomitant amitriptyline toxicity. Promazine was discontinued three days after admission. At follow-up approximately six months later, the patient remained off promazine and reported sustained symptom resolution and no further hospital attendances. This case highlights the importance of considering NMS in unexplained fever during antipsychotic treatment, conducting repeated medication reviews, and asking sensitive questions, regardless of a patient's professional healthcare background.

## Introduction

Neuroleptic malignant syndrome (NMS) is a rare but potentially fatal adverse reaction associated principally with dopamine-antagonist medication such as antipsychotics. Its characteristic features are pyrexia, altered mental state, severe muscle rigidity, and autonomic instability, frequently accompanied by raised creatine kinase (CK), leucocytosis, and rhabdomyolysis [1,2]. The presentation is variable; no single investigation is diagnostic, and the syndrome can resemble sepsis, central nervous system infection, serotonin toxicity, anticholinergic toxicity, and other neurological or metabolic disorders [1,3]. Unexplained fever in a patient receiving antipsychotics should therefore prompt consideration of NMS alongside urgent assessment for infection, particularly when rigidity, altered mental state, or autonomic changes are present [3].

This report describes a patient with recurrent episodes of fever, confusion, and muscle rigidity, with six hospital presentations over approximately 10 months and no cause identified despite extensive investigations. During the most recent admission, review of previous medical records, pharmacy records, and a thorough, non-judgemental discussion brought possible medication toxicity into focus. This patient was a healthcare worker, which may have influenced the way the history was taken and the patient was treated overall, potentially contributing to diagnostic delay, although this could not be established retrospectively from the available documentation.

This case report was previously presented as a poster at the 2026 Society for Acute Medicine Glasgow conference on October 8, 2026.

## Case presentation

At the most recent presentation, designated day 0 (D 0), a 51-year-old woman was brought to the emergency department by ambulance with a one-day history of fever, confusion, muscle rigidity, and shaking episodes. Her temperature was 39.4°C, heart rate was 126 beats/min, and blood pressure was 163/68 mmHg. Her medical history included anxiety, asthma, and chronic neuropathic pain. Prescribed medication included promazine 50 mg three times daily (first prescribed 12 months before this admission), amitriptyline 75 mg at night, diazepam, paracetamol, codeine, and asthma inhalers. She normally worked in healthcare but had not worked for several months because of significant physical and mental health difficulties.

She was asked about illicit substance use and possible medication overdose, both of which she denied. Empirical antibiotics were commenced for presumed sepsis of unknown origin in the emergency department, and she was referred to the medical team for further investigation and management. Blood tests showed mild neutrophilia and a mildly raised C-reactive protein (CRP) concentration of 21.5 mg/L. CK was 832 U/L when measured 24 hours after admission (D +1; Table 1). On admission, all of the patient's medication was continued at the doses specified on her repeat prescription, including promazine 50 mg three times daily. All presenting symptoms improved within 24 hours and had largely resolved by 72 hours (D +3).

**Table 1:** Blood test results during the most recent admission ↑ indicates an elevated result; ↓ indicates a reduced result. Reference intervals are reproduced from the laboratory reports where available; 'Not available' denotes an interval absent from the reviewed records. Creatine kinase was measured 24 hours after admission. The pH, bicarbonate, and lactate results are from venous blood gas analysis. Acute kidney injury classification follows Kidney Disease: Improving Global Outcomes criteria [4]; a laboratory stage of 0 is shown as 'no acute kidney injury.'

Test (unit)	Result	Reference range
White cell count (×10⁹/L)	9.5	3.7-11.0
Neutrophils (×10⁹/L)	7.9 ↑	1.7-7.5
Haemoglobin (g/L)	109 ↓	115-165
Platelets (×10⁹/L)	425	150-450
Total bilirubin (µmol/L)	9	1-21
Alanine aminotransferase (U/L)	21	0-33
Alkaline phosphatase (U/L)	73	20-130
Gamma-glutamyl transferase (U/L)	182 ↑	9-36
Prothrombin time (s)	12.2	9.4-12.5
International normalised ratio	1.1	Not available
Sodium (mmol/L)	134	133-146
Potassium (mmol/L)	3.9	3.5-5.3
Urea (mmol/L)	2.9	2.5-7.8
Creatinine (µmol/L)	75	44-97
Estimated glomerular filtration rate (mL/min/1.73 m²)	75	Not available
Acute kidney injury classification	No acute kidney injury	No acute kidney injury
C-reactive protein (mg/L)	21.5 ↑	0.0-10.0
Creatine kinase (U/L)	832 ↑	0-170
pH	7.43	7.35-7.45
Bicarbonate (mmol/L)	23.9	22.0-29.0
Lactate (mmol/L)	1	0.6-2.0

The patient reported similar episodes of varying but lesser severity occurring at home almost weekly. Review of medical records identified six hospital presentations, including the most recent admission, over approximately 10 months with no unifying cause established. This prompted a detailed review of previous presentations and documentation.

Review of her previous admissions revealed a particularly severe episode approximately six months before the most recent presentation (D -197), when she presented with extreme agitation, vomiting, muscle rigidity, a temperature above 40°C, and tachycardia of 164 beats/min. The admission electrocardiogram (ECG) demonstrated sinus tachycardia at 151 beats/min with a normal QRS duration but prolonged QT interval, with a Fridericia-corrected QT interval of 490 ms, calculated using Fridericia's formula [5] (Figure 1). The patient subsequently developed hypotension and shock requiring approximately 10 L of intravenous fluid replacement in the first 24 hours and vasopressor support in an intensive care unit. She was treated empirically for sepsis of unknown origin, including antibacterial and antiviral treatment for central nervous system infections such as meningitis and encephalitis.

**Figure 1:**
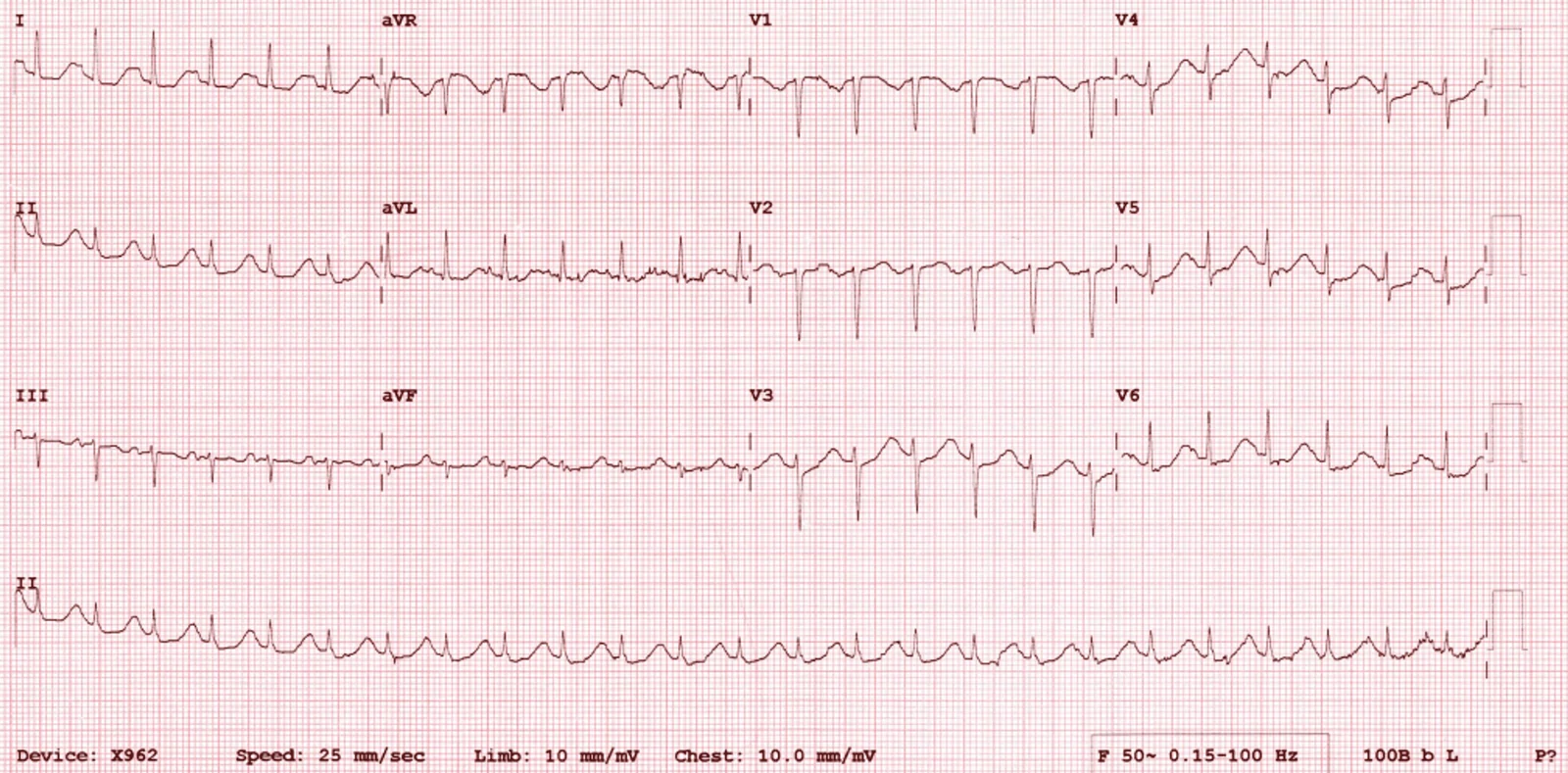
Admission electrocardiogram during the earlier severe episode Scanned reproduction of the patient's original printed 12-lead electrocardiogram demonstrating sinus tachycardia at 151 beats/min, a normal QRS duration, and a prolonged Fridericia-corrected QT interval of 490 ms [5]. Leads I, II, and III are standard bipolar limb leads; aVR, aVL, and aVF are the augmented limb leads (augmented voltage right arm, left arm, and foot, respectively); V1-V6 are the six chest leads. QT denotes the interval from the onset of the QRS complex to the end of the T wave; QRS denotes ventricular depolarisation.

In addition to the abnormal ECG, blood tests showed substantial metabolic abnormalities. The patient had profound mixed metabolic and respiratory alkalosis, likely related to vomiting and hyperventilation. The white cell count was 24.9×10⁹/L and lactate was 5.1 mmol/L; these findings prompted treatment for sepsis of unknown origin despite a normal CRP concentration. CK was markedly elevated on admission at 7272 U/L (Table 2).

**Table 2:** Blood test results during the earlier severe admission ↑ indicates an elevated result; ↓ indicates a reduced result. Reference intervals are reproduced from the laboratory reports where available; 'Not available' denotes an interval absent from the reviewed records. The pH, partial pressure of carbon dioxide, bicarbonate, chloride, and lactate results are from venous blood gas analysis. The recorded stage 1 acute kidney injury is reported using the Kidney Disease: Improving Global Outcomes classification [4].

Test (unit)	Result	Reference range
White cell count (×10⁹/L)	24.9 ↑	3.7-11.0
Neutrophils (×10⁹/L)	20.2 ↑	1.7-7.5
Haemoglobin (g/L)	154	115-165
Platelets (×10⁹/L)	510 ↑	150-450
Total bilirubin (µmol/L)	27 ↑	1-21
Alanine aminotransferase (U/L)	82 ↑	0-33
Alkaline phosphatase (U/L)	44	20-130
Gamma-glutamyl transferase (U/L)	104 ↑	9-36
Prothrombin time (s)	11.9	9.4-12.5
International normalised ratio	1.1	Not available
Sodium (mmol/L)	133	133-146
Potassium (mmol/L)	3.0 ↓	3.5-5.3
Urea (mmol/L)	8.7 ↑	2.5-7.8
Creatinine (µmol/L)	125 ↑	44-97
Estimated glomerular filtration rate (mL/min/1.73 m²)	41 ↓	Not available
Acute kidney injury classification	Stage 1	No acute kidney injury
C-reactive protein (mg/L)	1.9	0.0-10.0
Creatine kinase (U/L)	7272 ↑	0-170
pH	7.60 ↑	7.35-7.45
Partial pressure of carbon dioxide (kPa)	4.6	Not available
Bicarbonate (mmol/L)	34.4 ↑	22.0-29.0
Chloride (mmol/L)	90 ↓	98-107
Lactate (mmol/L)	5.1 ↑	0.6-2.0

After two days of empirical and supportive treatment (D -195), the fever had resolved, vasopressors were no longer required, and the patient was fully alert and orientated. Symptoms had fully resolved five days after admission (D -192), but no unifying cause was established. Available additional investigations are summarised in Table 3. Blood cultures showed no growth after two days' incubation. Cerebrospinal fluid (CSF) was clear and colourless, with normal white cell count, protein and negative culture, and polymerase chain reaction tests for the listed pathogens. CSF glucose and lactate were mildly above the laboratory intervals; the glucose was interpreted alongside a paired serum glucose of 7.5 mmol/L (CSF/serum ratio 0.63).

**Table 3:** Additional microbiological, cerebrospinal fluid, autoimmune, and thyroid investigations ↑ indicates a result above the laboratory interval. Reference intervals are reproduced from the laboratory reports where available; expected adult CSF cell counts are from Hrishi and Sethuraman [6]. 'Not available' denotes an interval absent from the reviewed records. AI, antibody index; CSF, cerebrospinal fluid; IgG, immunoglobulin G; PCR, polymerase chain reaction.

Investigation (unit)	Result	Reference/expected value
Antinuclear antibody screen	Negative	Negative
Anti-myeloperoxidase antibody (AI)	<0.2	0.0-0.9
Anti-proteinase 3 antibody (AI)	<0.2	0.0-0.9
Complement C3 (g/L)	1.48	0.70-1.80
Complement C4 (g/L)	0.22	0.12-0.36
Rheumatoid factor (IU/mL)	4.6	Not available
Anti-cyclic citrullinated peptide IgG antibody (kU/L)	<0.6	Not available
Free thyroxine (pmol/L)	23.4 ↑	10.0-22.1
Thyroid-stimulating hormone (mU/L)	0.98	0.10-4.00
Blood culture	No growth at 2 days	No growth
CSF appearance	Clear, colourless	Clear, colourless
CSF white cell count (×10⁶/L)	<1	0-5 [6]
CSF red cell count (×10⁶/L)	3	0 [6]
CSF protein (g/L)	0.37	0.15-0.45
CSF glucose (mmol/L)	4.7 ↑	2.2-4.4
Paired serum glucose (mmol/L)	7.5	Not available
CSF/serum glucose ratio	0.63	Approximately two-thirds [6]
CSF lactate (mmol/L)	2.9 ↑	1.3-2.5
CSF Gram stain	No organisms seen	No organisms seen
CSF culture	No growth	No growth
CSF bacterial PCR: *Neisseria meningitidis* (including group B) and *Streptococcus pneumoniae*	Not detected	Not detected
CSF viral PCR: herpes simplex types 1 and 2, varicella zoster, enterovirus, and parechovirus	Not detected	Not detected

Computed tomography (CT) of the head and magnetic resonance imaging (MRI) of the brain and spine did not identify a unifying structural or infective cause. Representative axial CT head, axial T2-weighted MRI brain, and sagittal T2-weighted MRI spine images are shown in Figures 2-4, respectively. The spinal images showed no evidence of discitis.

**Figure 2:**
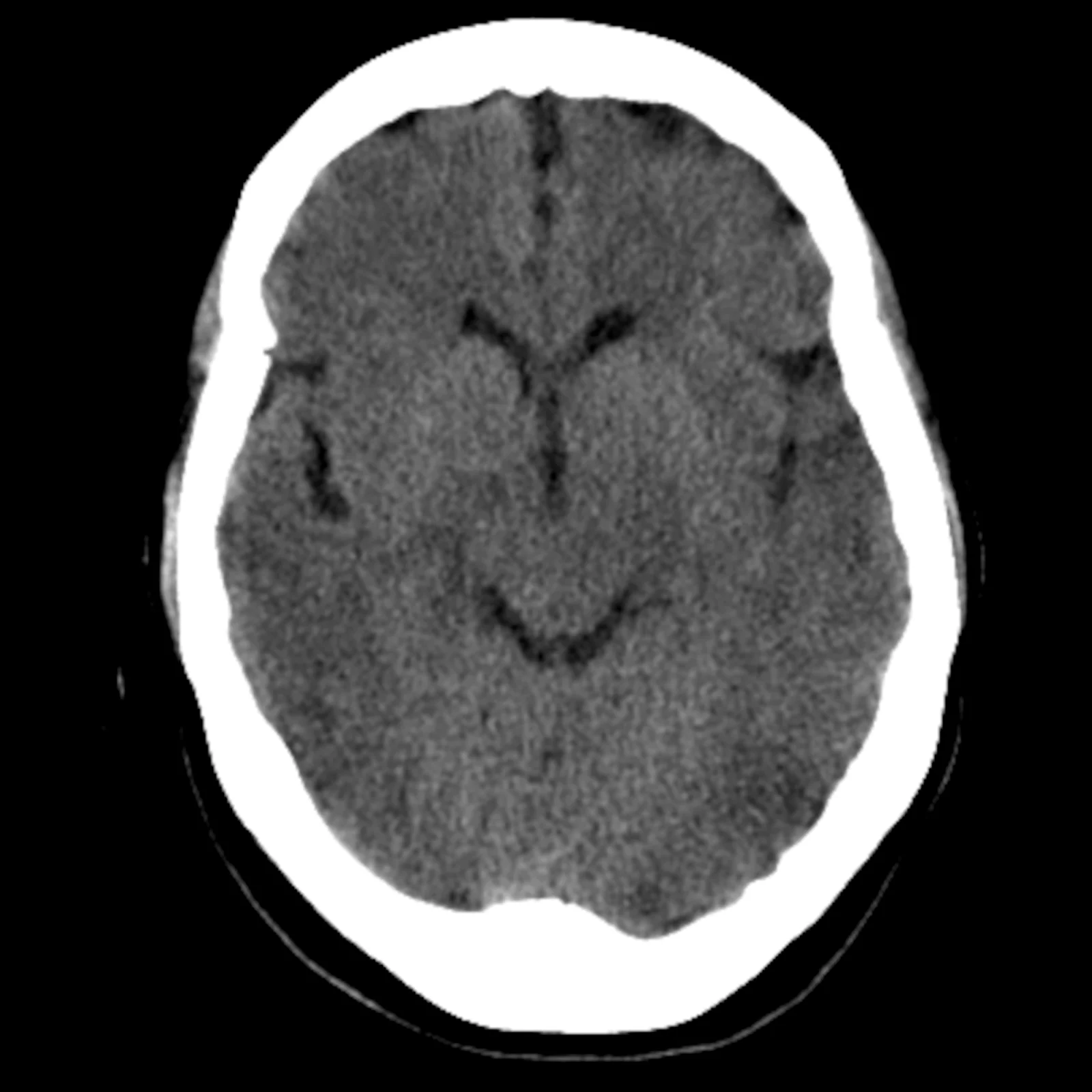
Axial computed tomography of the head Axial computed tomography image of the head displayed in a brain (soft tissue) window, demonstrating normal intracranial appearances with no acute abnormality identified.

**Figure 3:**
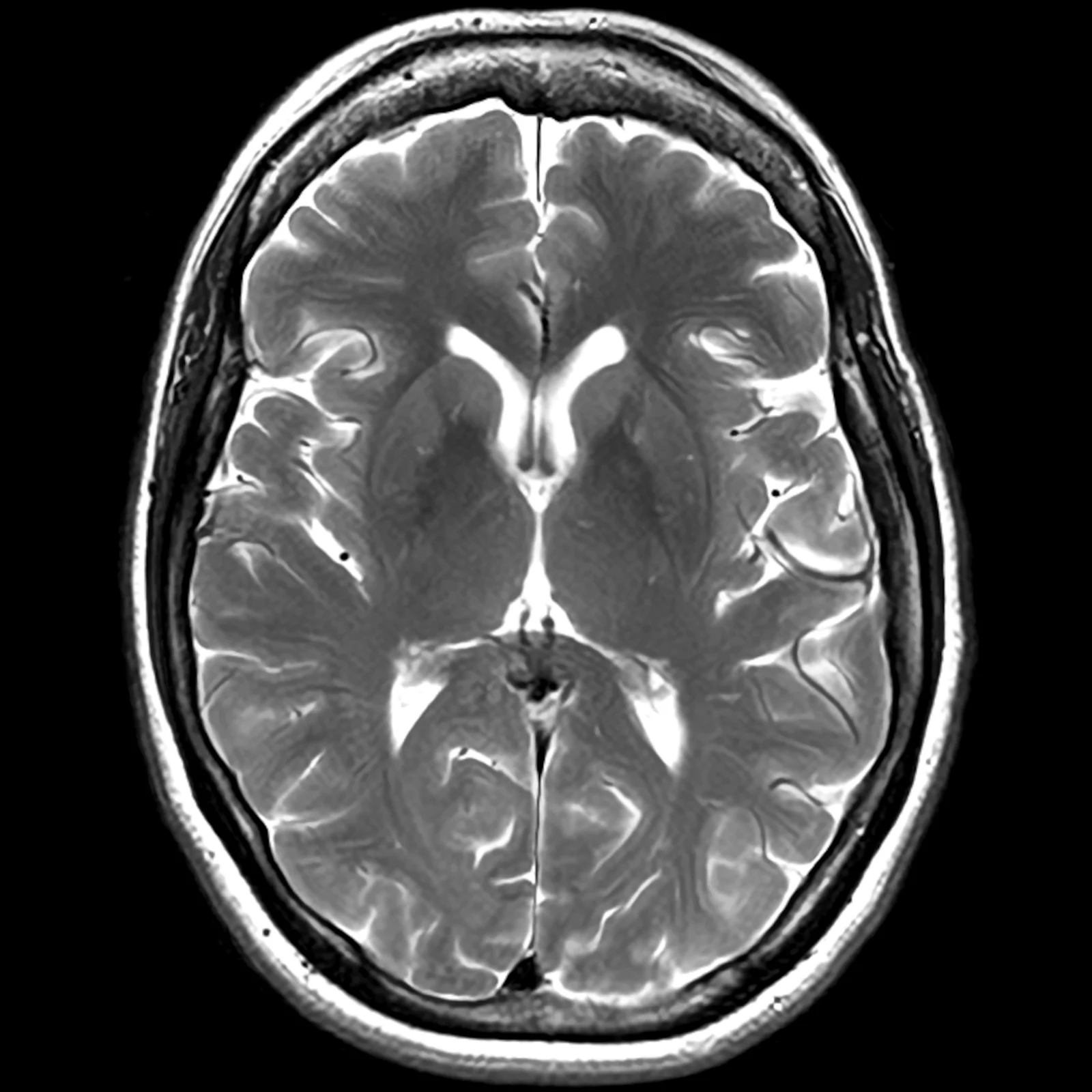
Axial T2-weighted magnetic resonance imaging of the brain Axial T2-weighted magnetic resonance image of the brain demonstrating normal appearances with no abnormality identified on the displayed slice.

**Figure 4:**
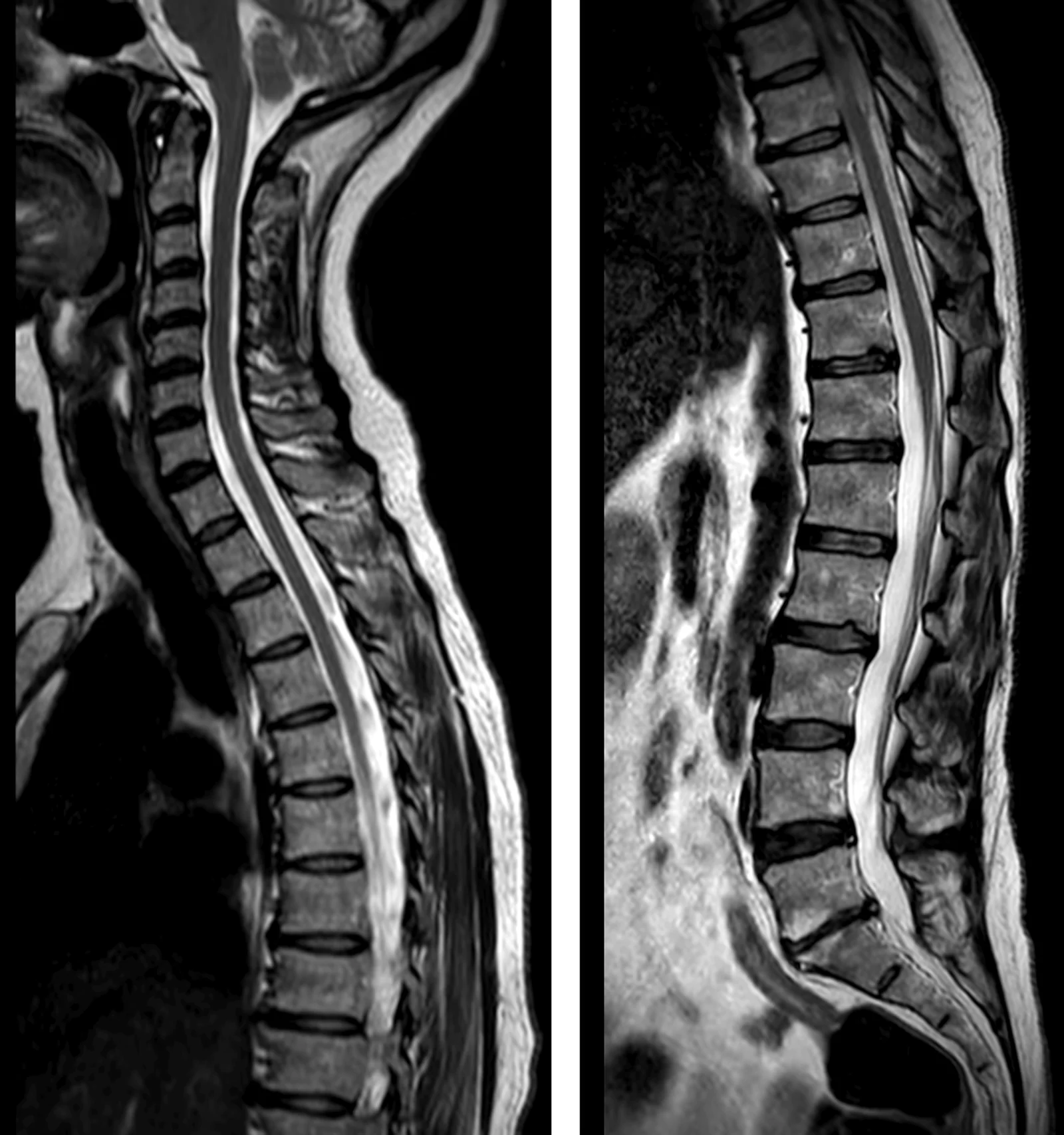
Sagittal T2-weighted magnetic resonance imaging of the spine Sagittal T2-weighted magnetic resonance images showing the cervicothoracic spine from C1 to T9 (left) and the thoracosacral spine from T7 to S5 (right). Both images demonstrate normal spinal appearance with no evidence of discitis. C, T, and S denote cervical, thoracic, and sacral vertebral levels, respectively. The two images were combined into a single figure using GIMP 3.2; no modifications were made to the image content.

Routine systemic autoimmune screening was negative, including the antinuclear antibody screen and antibodies to myeloperoxidase and proteinase 3; complement concentrations were within the reported laboratory intervals. Free thyroxine was mildly elevated with thyroid-stimulating hormone within range. Rheumatology and neurology reviews did not identify a unifying diagnosis. Autoimmune encephalitis, mitochondrial disease, and acute porphyria were considered, but autoimmune encephalitis antibody testing and porphyria screening were not performed.

Alongside this unrevealing diagnostic work-up, previous medical records contained several clues to possible medication toxicity. One previous entry mentioned a possible co-codamol overdose the preceding year, and an ambulance attendance record documented a relative's concern that the patient had possibly taken an overdose. The patient was asked about a possible medication overdose during an earlier admission, but this possibility was not explored further at the time. A further clue was improvement during hospital care, usually within 24-72 hours, with recurrence after discharge. Resolution took longer in some episodes, including one in which low-grade fever persisted for seven days.

Returning to the most recent admission, these findings prompted a further discussion with the patient on D +3, approximately 72 hours after presentation, by which time symptoms had again largely resolved. The patient was asked again about her mental health, the circumstances surrounding the previous suspected overdose and whether she might have taken more medication than prescribed. Understandably, the patient initially found the discussion difficult and upsetting. However, time was allowed to explore concerns and establish rapport, and eventually the patient reported that she might have taken additional medication, although she never clearly specified which preparation she had taken or how much. The patient found the discussion distressing, and the exact quantities taken were not established, as further clarification was not expected to alter management at that stage. She reported low mood but denied suicidal intent, explaining that the additional medication had been taken to improve sleep and relieve neuropathic pain. As such, she did not view taking additional medication for symptom relief as an overdose in the traditional sense.

Further review of pharmacy records showed that six months' worth of promazine prescriptions had been approved and dispensed during the preceding three months. This established increased access to medication but did not establish the quantity ingested. Promazine is a typical antipsychotic associated with NMS [3,7]. Taken together, the clinical pattern, previous concerns about overdose, the patient's account, and the prescription history led to a working diagnosis of probable recurrent NMS associated with possible excess promazine use. Possible concomitant amitriptyline toxicity was also considered.

The patient was referred to the mental health team for assessment and review of her treatment. Following the working diagnosis of probable NMS on D +3, promazine was discontinued and additional support was offered. At follow-up on D +183, approximately six months after the most recent presentation, the patient remained off promazine, with no further medication changes. She reported sustained symptom resolution and no further hospital attendances; the latter was supported by the hospital records. The presentations, management, and follow-up are summarised in Table 4.

**Table 4:** Timeline of presentations, management, and follow-up D 0 denotes the most recent presentation; negative and positive day numbers indicate days before and after this presentation, respectively. Attendance rows include the subsequent course of that admission. CK, creatine kinase; CNS, central nervous system; NMS, neuroleptic malignant syndrome.

Hospitalisation	Day	Presentation or milestone	Management and outcome
1	D -299	Hypokalaemia; temperature 37.6°C	Supportive management only; symptoms resolved within 24 hours
2	D -197 to D -192	Severe presentation: fever, confusion, rigidity, hypotension, and raised CK	Intensive care, vasopressors, and approximately 10 L intravenous fluids. Treated for suspected CNS infection; investigations were unrevealing. Improved after two days; discharged after five days without a clear diagnosis. Symptoms resolved
3	D -83	Hypokalaemia; temperature 38.2°C	Treated for infection of unknown source; symptoms resolved within 72 hours
4	D -71	Temperature 38.5°C	Treated for infection of unknown source. Mild pyrexia persisted for seven days; symptoms subsequently resolved
5	D -34	Temperature 37.6°C	Broad-spectrum antibiotics; symptoms resolved within 48 hours
6	D 0	Most recent presentation: fever, confusion, and rigidity	Initially treated for suspected infection
6	D +1	CK measured at 832 U/L	Presenting symptoms had improved within 24 hours
6	D +3	Further patient review and medication history	Probable promazine-associated NMS recognised; promazine discontinued
—	D +183	Follow-up contact	Remained off promazine; no further attendances; patient reported sustained symptom resolution

## Discussion

In retrospect, the earlier severe presentation showed many recognised features of NMS, particularly the combination of high fever, altered mental state, muscle rigidity, autonomic instability, and markedly raised CK (Table 5) [1,2]. The absence of an alternative explanation, subsequent disclosure of excess medication use, and evidence of increased access to promazine supported the working diagnosis of NMS. Ultimately, it was not possible to establish exactly which medication had been taken or how much, so the diagnosis remained probable rather than confirmed at the time.

**Table 5:** Key clinical and laboratory features of neuroleptic malignant syndrome Key features as summarised from Oruch et al. and Grover et al. [1,2]. No single finding is diagnostic.

Feature	Typical finding
Hyperthermia	Very high fever
Muscle rigidity	Generalised increased muscle tone, often described as lead-pipe rigidity
Altered mental state	Agitation, confusion, delirium, or reduced consciousness
Tachycardia	Raised heart rate
Autonomic instability	Elevated, reduced, or fluctuating blood pressure
Tachypnoea	Raised respiratory rate
Diaphoresis	Excessive sweating
Tremor	Involuntary shaking
Elevated creatine kinase	Muscle injury; rhabdomyolysis may occur
Leucocytosis	Raised white cell count

Published reports illustrate how incomplete presentations can delay recognition. Collins et al. described confusion, rigidity, autonomic instability, and raised CK, with recognition of NMS delayed by the absence of high fever [8]. Kutumbaka et al. reported NMS without rigidity, complicated by ileus and severe renal injury [9]. Our patient's earlier severe episode included both hyperthermia and rigidity; the difficulty lay in linking recurrent episodes of variable severity with medication exposure. Rapid inpatient improvement was a useful clue to possible medication toxicity but was not diagnostic of NMS.

Although promazine was not discontinued until D +3, inpatient administration was supervised and limited to the prescribed dose of 50 mg three times daily. If the patient had been taking additional promazine before admission, this would have represented a reduction in exposure despite continuation of the prescribed dose. Improvement during hospitalisation was therefore compatible with cessation of possible excess dosing. However, the preparation and quantity reportedly taken in excess could not be established, so this interpretation remained inferential.

Promazine has also been implicated in a report of NMS and rhabdomyolysis after a dose increase in a patient receiving other antipsychotics and carbamazepine [7]. Such polypharmacy limits attribution to one agent. Similarly, the dispensing history in this case supports access to additional promazine but cannot prove excessive ingestion or establish that every recurrence represents NMS.

NMS is usually triggered by dopamine receptor antagonism, particularly from antipsychotics. Its pathophysiology remains incompletely understood; proposed mechanisms include central dopamine D2 receptor blockade affecting thermoregulation and motor control, and abnormal skeletal muscle calcium handling contributing to rigidity and heat production [10]. Dose escalation, dehydration, and agitation may increase risk, although NMS can occur during apparently stable treatment [3,10].

Early recognition should prompt immediate withdrawal of the suspected causative drug and supportive management, including cooling, intravenous fluids, correction of electrolyte abnormalities, and monitoring for rhabdomyolysis, renal injury, and cardiac complications, with intensive care support when required [2,3]. Benzodiazepines may help control agitation. Bromocriptine, amantadine, or dantrolene may be considered in severe or persistent cases, and electroconvulsive therapy in refractory cases; evidence for specific treatments is largely based on case reports, case series, and expert opinion [2,10].

Anticholinergic toxicity possibly contributed to the presentation because both amitriptyline and promazine possess antimuscarinic activity. Both anticholinergic toxicity and NMS may cause fever, tachycardia, agitation, and confusion [2,11]. However, generalised severe rigidity, high fever, and profound CK elevation favour NMS. Anticholinergic toxicity more typically produces hot, dry skin, dry mucous membranes, mydriasis, urinary retention, and reduced bowel sounds, and marked rigidity is not characteristic. Severe physical agitation or seizures could cause secondary rhabdomyolysis [2,11]. In this case, extreme agitation was documented during the severe presentation.

Amitriptyline toxicity may additionally produce potentially lethal neurological and cardiovascular effects. Antimuscarinic manifestations can be accompanied by seizures, hypotension, conduction abnormalities, QT prolongation, and ventricular dysrhythmias [12,13]. The earlier ECG showed a prolonged corrected QT interval with a normal QRS duration. This provided no evidence of marked intraventricular conduction delay on that tracing but did not exclude concomitant amitriptyline toxicity [12].

Several signs of anticholinergic toxicity, such as dry skin or mydriasis, are subtle and may not have been documented consistently across admissions, which limits retrospective differentiation. Other relevant examination findings, including urinary retention, clonus, and hyperreflexia, were not explicitly documented; it is therefore unclear whether these findings were present or absent. Overall, the patient's symptoms were suggestive of NMS, potentially overlapping with amitriptyline toxicity, although the available records did not allow a definitive distinction.

The unrevealing investigations should also be interpreted with caution. Absence of CSF pleocytosis and normal brain MRI do not exclude autoimmune encephalitis [14]. Specific autoimmune encephalitis antibody tests and porphyria screening were not performed, so these conditions were not definitively excluded. The clinical course and medication history favoured a drug-related explanation, while the retrospective nature of the review and uncertain ingestion limited diagnostic certainty.

Initial empirical treatment for sepsis was appropriate because sepsis and central nervous system infection were potentially life-threatening differential diagnoses. However, recurrent presentations, multiple negative investigations, rapid improvement during hospital admission, and repeated indications of possible overdose should have triggered repeat assessment and consideration of a medication toxidrome. There was considerable emphasis on infection and rare inflammatory or metabolic disorders, yet history and medication review received comparatively little attention. Considering NMS alongside infection allows medication withdrawal and complication monitoring to begin without waiting for every alternative investigation to be completed [2,3].

The patient's professional background may have affected the clinical interaction. In a qualitative study of clinicians caring for healthcare professional patients, care was described as prioritised, but at the same time there was reluctance to ask personal or emotionally challenging questions. Participants reported greater commitment or privileges for some professional patients, while also describing intimidation and ambiguity about whether the individual should be approached primarily as a patient or as a professional colleague [15]. In this case, much of the history and information reported by the patient were taken at face value without further critical evaluation. Whether professional status contributed to delay in this case remains uncertain, but reluctance to explore uncomfortable topics may impede a complete medication and mental health history.

The patient eventually provided the crucial clue herself, but it was only after a prolonged, non-judgemental conversation in which the distinction between intentional self-harm and taking additional medication for symptom relief could be safely explored. This illustrates the importance of repeat assessment and diagnosis review when evidence does not add up or does not explain the presentation. Finally, sensitive questioning about mood, suicidal intent, self-medication, and access to medicines should be undertaken consistently, irrespective of a patient's professional healthcare background.

## Conclusions

Unexplained fever in patients receiving antipsychotics should prompt consideration of NMS alongside urgent assessment for infection, particularly when accompanied by rigidity, altered mental state, or autonomic instability. In this case, review of previous records and time for a thorough, non-judgemental discussion led to a working diagnosis of probable promazine-associated NMS, with possible concomitant amitriptyline toxicity. This interpretation was further supported by the absence of further episodes once promazine was discontinued. The case was a reminder that patients may not describe medication overuse for symptom relief as an overdose. Finally, healthcare professionals-patients need the same systematic and objective assessment as other patients when being assessed by clinicians.
